# Escalation of an Otherwise Curable Cancer: Retroperitoneal Mass Destruction Associated With Testicular Seminoma in a Psychiatric Patient

**DOI:** 10.7759/cureus.10925

**Published:** 2020-10-13

**Authors:** Krishna Teja Challa, Megan LeBlanc, Habtemariam Makonnen

**Affiliations:** 1 Department of Medicine and Research, Avalon University School of Medicine, Willemstad, CUW; 2 Department of Medicine and Research, A.T. Still University of Health Sciences-Kirksville College of Osteopathic Medicine, Missouri, USA; 3 Department of Internal Medicine, St. Luke's Hospital and Mountain Vista Medical Centre, Phoenix, USA

**Keywords:** seminoma, psychiatric illness, chemotherapy, retroperitoneal mass, substance abuse

## Abstract

We report the case of a 31-year-old male patient with underlying psychiatric illness and substance abuse who presented to the emergency department with a chief complaint of abdominal pain. CT scan of the abdomen revealed a large retroperitoneal mass wrapping around the aorta and obstructing the left ureter causing hydronephrosis. Physical examination found a painless left testicular mass. The ultrasound revealed a left scrotal mass measuring 32 x 24 x 16 mm with evidence of increased vascularity and calcifications. The patient underwent ureteral stent placement and left testicular orchiectomy with the pathology evaluation revealing seminoma. Although follow-up care plans were made, the patient relapsed IV heroin abuse causing failure to attend oncological treatment appointments. Two months later upon readmission, a repeat of the CT scan of the abdomen and pelvis showed a persistent large left retroperitoneal mass with an increase in size and significant mass effect. The patient would be ultimately considered for hospice if lack of compliance were to continue for his chemotherapy, along with his comorbid underlying substance abuse and psychiatric illness. This case highlights the effect of mental illness on medical care and treatment, demonstrating how a treatable malignancy may result in greater morbidity and mortality in psychiatric populations.

## Introduction

While testicular cancers only make up 1% of all cancers in men, it is known to be the most common solid tumor type affecting men aged 15-35, with germ cell tumors such as seminoma comprising 95% of these. It is simultaneously known to be one of the most curable neoplasms, as well. The incidence in the United States, like many other parts of the world, has been increasing since the mid-20th century and from 1998-2011, census data showed the highest incidence of 6.57 in every 100,000 per year in non-Hispanic white men presenting with testicular germ cell tumors [1]. Although seminoma can present in more than one manner, the most common initial presentation is noticing a painless nodule or growing of a unilateral testicle [2]. However, a presentation with metastatic disease, including both regional and distant, can make up to fifteen percent of pure seminomas; in which case, the median five-year survival rate drops from 99% to 74%. Signs of malignancy may include but are not limited to neck mass, cough/dyspnea, bone pain, unilateral lower extremity swelling, anorexia, nausea, and/or vomiting. Late presentation can occur for a myriad of different reasons. Common causes are a lack of noticeable symptoms during the early disease state, disinclination to investigate testicular masses, lack of self-examination precision, or as the case was for this patient, lack of access to care, along with a misunderstanding of the mass’ significance [3-5]. Despite the potentially large variance in presenting symptoms, there is still no recommended screening for testicular cancer. Testicular self-examination stands at a class D recommendation by the United States Preventive Services Task Force (USPSTF) at primary care visits and is only recommended for select circumstances since there is no indication to date that it improves outcomes [6-7]. These exceptions include symptomatic men with potential regional or distal symptoms (testicular mass, neck or abdominal mass, gynecomastia, etc.), surveillance in those with a history of germ cell tumor, and those with a history of cryptorchidism, who have a higher overall lifetime risk of developing testicular cancer later in life [8].

This patient demonstrates a case of how an otherwise likely curable seminoma escalated to a large retroperitoneal mass causing left-sided hydronephrosis and mass effect, likely related to accompanying factors such as psychiatric illness, low socioeconomic status (SES), and other comorbidities. This case will discuss not only the specifics of his clinical course and germ cell tumor findings, but also the complex confounding factors that changed the course of diagnosis, treatment, and prognosis. These include a history of polysubstance abuse, psychiatric illness, previous/recent incarceration, low SES/homelessness, and lack of consistent medical care, among others. It is important to examine these factors because according to the National Survey of Drug Use and Health (NSDUH) in 2018, the incidence of substance abuse was over 19.7 million American adults [9], and according to the NIMH statistics in 2019, the incidence of severe mental illness (SMI) was approximately 4.5% of all US adults equating to over 11.2 million [10]. With these comorbidities being highly prevalent in society, it is important to examine their effect on health outcomes, patients, and our healthcare system.

## Case presentation

This case reports on a 31-year-old non-Hispanic white male who presented to the local hospital emergency department with severe abdominal pain, myalgias, chills, and headache as a transfer from the medical facility at his center for incarceration. Abdominal pain was rated eight out of 10 in severity, diffuse in nature, and associated with sharp, left-sided, localized back pain both of which began two days ago without any inciting incident. On palpation of the abdomen, a hard mass was noticed, which prompted a CT scan of the abdomen and pelvis, revealing a large retroperitoneal mass wrapping around the aorta and obstructing the left ureter causing hydronephrosis. The patient was given Tylenol 650mg, Morphine 8mg, and Fentanyl 25mg for pain and was referred to a specialized facility where this pain was also addressed with Hydromorphone 0.5mg Q4h PRN. The patient also had a significant psychiatric history including major depressive disorder, bipolar disorder type 1, attention deficit hyperactivity disorder as well as a five-year heavy polysubstance abuse including IV heroin and methamphetamine, and nicotine. At first presentation, the patient had not used heroin, alcohol, or tobacco products since beginning his current incarceration. The reports were unclear about how compliant his bipolar and major depressive disorder treatments were in prison and thereafter. His treatment history included five sessions of electroconvulsive therapy treatment for major depressive disorder, as well as prescribed Bupropion for depression, Guanfacine for ADHD, and Seroquel for bipolar and depression. Additional medical history included untreated hepatitis C likely due to IV drug abuse (IVDA). A more complete history of presenting illness revealed a lack of vomiting, cough, shortness of breath, chest pain, or dyspnea both on exertion and at rest. The patient also denied hematochezia or melena and reported normal bowel movements without diarrhea or constipation. Patient noted a decreased urinary stream without dysuria, hematuria, or change in frequency. A review of systems revealed unintentional 50 lb weight loss in the last six months. Physical examination revealed costovertebral angle tenderness on the left and +1 lower extremity edema bilaterally. Genitourinary exam revealed a non-mobile mass with soft tissue swelling of the left testicle. The patient later admitted to the presence of painless testicular mass for 1.5 years, which he stated he thought was normal. So although this mass was previously noticed by the patient, diagnosis and treatment were delayed ultimately allowing for a minimum of 1.5 years of disease progression.

**Figure 1:**
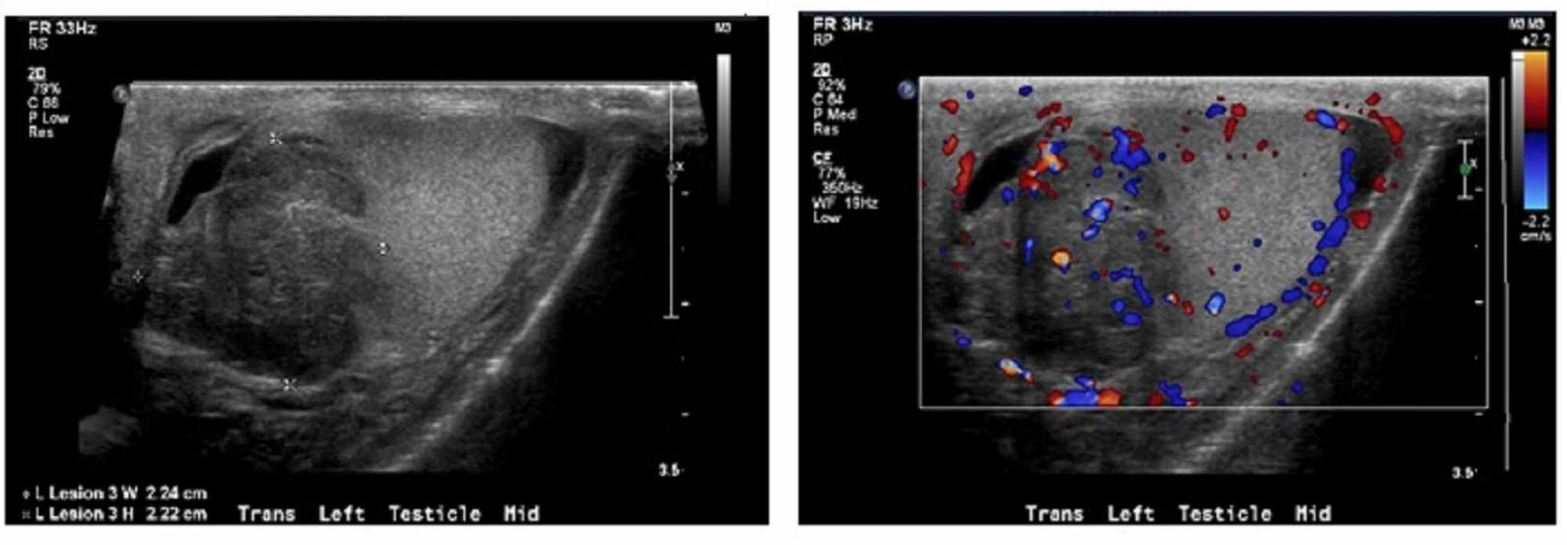
Hypervascular mass in the left testis measuring 35 x 22 x 22 mm

**Figure 2:**
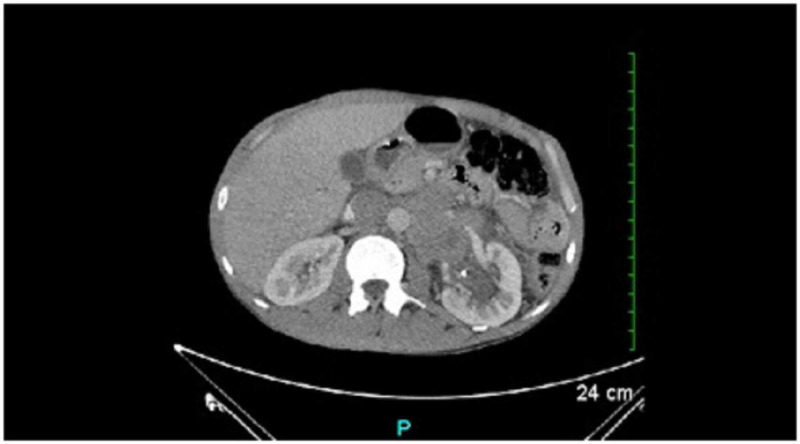
CT scan of the abdomen showing retroperitoneal mass measuring 4.7 x 6.3 x 7 cm and ureteral stent

Objective findings included lab results demonstrating low hemoglobin of 6.3g/dl, mean corpuscular volume (MCV) of 85, reticulocyte count at 3%, low iron at 25mcg/dL, total iron binding capacity (TIBC) at 211 mg/dl, and normal ferritin at 117 ng/mL, thus leading to suspect anemia of chronic disease. Serum markers were also measured and Beta-human chorionic gonadotropin (B-hCG) was found to be nine. This represented an abnormal presence of B-hCG secreted from cancer cells transforming into syncytiotrophoblasts, indicating testicular cancer. Abnormal Lactate Dehydrogenase (LDH) of 317 U/L was also revealed suggesting tissue damage, and finally, Alpha Feto Protein (AFP) was found to be within normal limits. With a mild Beta-hCG elevation, a larger increase in LDH, and AFP in normal limits, along with histologic typing of seminoma, it was diagnosed as a seminoma. This serum marker combination effectively rules out differential diagnoses including yolk sac tumors, choriocarcinoma, teratoma, and embryonal carcinoma. Although the pathology report did not include and/or rule out anaplastic seminoma, this would not have any effect on treatment or prognosis, as therapeutic response and survival are not unfavorably affected by an increase in mitotic figures [11]. CT scan of the abdomen and pelvis with contrast and ultrasound of the testis were both ordered. Ultrasound of the testis revealed left scrotal mass with evidence of increased vascularity and calcifications (Figure 1) at which time urology and oncological consults were requested. On urology consult, radical left orchiectomy of the testis was planned due to concern of malignancy and left ureteral stent placement was indicated by the hydronephrosis of the left kidney associated with the retroperitoneal mass. The patient underwent both procedures without complication or adverse incident. CT scan of the abdomen and pelvis showed the retroperitoneal mass and left ureteral stent (Figure 2). After left radical orchiectomy, the testicle was preserved for examination, and pathology report of the testicular specimen revealed seminoma with pT2, NX staging. A metastatic evaluation was performed due to findings of cough, bilateral leg swelling, anorexia, nausea, and vomiting. This included chest, abdomen, and pelvic CT imaging as well as lower extremity duplex ultrasound. Chest CT showed bibasilar atelectasis, partial visualization of left hydronephrosis, and a few small mediastinal nodes present, which radiology report states may be reactive. Abdominal CT findings were previously mentioned. Pelvic CT demonstrates small mesenteric lymph nodes, as well as bilateral inguinal and pelvic lymph nodes. Bilateral lower extremity duplex ultrasound was found to be normal. The patient was suggested to undergo adjuvant chemotherapy with Bleomycin, Etoposide, and Cisplatin as postoperative prophylaxis. Unfortunately, the patient relapsed with his IVDA and began using heroin. This ultimately led to failure to follow up with the oncologist for chemotherapy and continuation of treatment.

**Figure 3:**
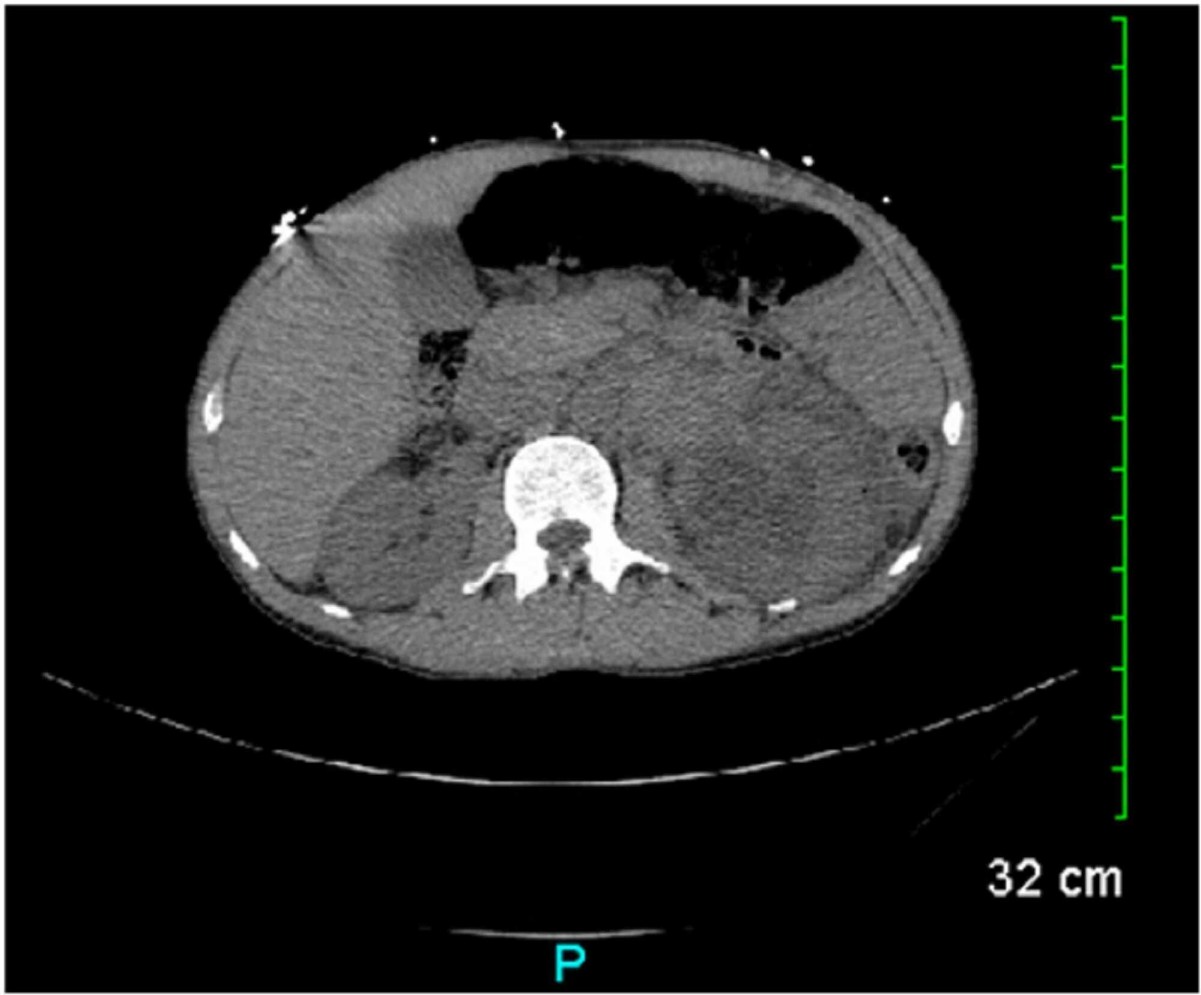
CT scan of the abdomen revealing central necrotic mass measuring 7 x 5.4 x 9 cm with mass-effect

Two months later the patient was referred to this facility again, from a psychiatry inpatient unit where he had checked in for heroin detox. During this two-month time frame, he had been released from the aforementioned incarceration, relapsed heroin IVDA, and became homeless. At the time of this second hospital admission, CT scan of the abdomen and pelvis with contrast was again indicated. This revealed a persistent, large, left retroperitoneal, central necrotic mass with measurement showing approximately 64% increase in size, resulting in mass-effect with displacement of the left renal vein, narrowing of the left renal artery without occlusion, and compression of the left ureter causing hydronephrosis (Figure 3). The mass has increased in size compared to the previous measurement of 4.7 x 6.3 x 7 cm over the course of only two months. Though this is a rapid growth rate compared to a typically reported slower-growing germ cell tumor malignancy, it is extremely common to see necrosis in retroperitoneal seminoma masses in up to 80% of patients who have them, as well as for these masses to be entangled within the great vessels of the abdomen [12]. The patient would be ultimately considered for hospice if lack of compliance were to continue for his chemotherapy, along with his comorbid underlying substance abuse and psychiatric illness.

## Discussion

This case report emphasizes patient awareness regarding testicular abnormalities and how psychiatric illness comorbidities in a patient can affect health outcomes. Testicular self-exam (TSE) is an easy palpatory procedure of the testes that the patient can do himself regularly to detect unusual lumps and bumps within his testicles. As previously mentioned, the USPSTF gives a class D recommendation for screening of testicular cancer, and currently, there is a grade 2C recommendation against having patients utilize TSE themselves since there is low sensitivity and specificity and insufficient evidence at this time that it improves patient outcomes. However, there is clear evidence that catching primary testicular cancer before metastasis maintains the 99% cure rate, whereas after metastasis this drops to 74% [3]. For this reason, regular utilization of TSE can make improvements in preventing a late-stage diagnosis of testicular cancers [13]. Although this patient had been aware of a mass in his testicle for over a year and thus could have been worked up significantly earlier than he was, not knowing that the finding was abnormal delayed his diagnosis. This addresses a large gap in public understanding regarding TSE and testicular cancer [14]. Though this topic is widely debated and recommendations differ around the world, including recommendations against the use of TSE by many US medical society guidelines, this case demonstrates a profound example of the importance of early detection.

Another specific barrier to this particular case was his history of mental illness. In this case, there are both provider and patient-related factors standing in the way of appropriate patient care. Druss et al. addresses these problems and defines them to include physical separation of medical & psychiatric facilities, fragmentation of care between specialties, muddled clarity of who is in charge, navigating combination treatments between medicine and psychiatry, severe deficiency of access to outpatient care in low SES populations, and overall scarcity of resources [15-16]. This was seen very directly at the patient's second presentation as a transfer from an adult psychiatry inpatient detox facility. Despite his need for psychiatric care and detox from IV heroin, providers were forced to choose whether to address his medical versus psychiatric care, despite a clear need for a marriage of the two. Additionally, as an IV drug abuser of opiates and amphetamines, he fits another common issue where providers view somatic medical complaints as drug-seeking or otherwise psychological symptoms [16]. This is unfortunately also a frequent cause of undertreating cancer pain in this population. In addition to the psychiatric comorbidities this patient already possessed, there are also found to be psychosocial and sexual difficulties for men with testicular cancer, noting a near 10% who experience these challenges on a long-term basis [17]. This has been noted to stimulate anxiety, depression, and interfere with interpersonal relationships for these men, even in those who may not have had a prior issue, and likely exacerbating issues in those who have prior diagnoses.

In this patient, severe mental illness (SMI) including bipolar, major depressive disorder, ADHD, and polysubstance addiction have all contributed to a lack of treatment compliance on the part of the patient and a discontinuity of care on behalf of physicians. Despite a treatment plan made with oncology at first diagnosis, the patient relapsed to IVDA and failed to attend outpatient chemotherapy appointments and further treatment. He appeared to be a poor medical historian on numerous occasions and lacked the responsibility needed to maintain a regimented treatment schedule. Unfortunately, this could ultimately lead to a consideration of hospice if the patient continued to disregard healthcare plans made. This entanglement of psychiatric comorbidities with the oncological disease has worsened and complicated his disease course to the point of severe protein malnutrition and possible submission to disease with hospice. This patient continues to fight his cancer at this time and has brought to light many pertinent shortcomings of healthcare capability in his and similar psychiatric patients.

## Conclusions

We have addressed shortcomings within the care of this case that apply more broadly to coordinated healthcare. Firstly, preventing delayed diagnosis, patient compliance, and general public awareness for risk factors and early symptomology are all undeniably crucial elements for positive outcomes both in testicular cancers, as well as with any disease. Secondly, the multifactorial nature of inequalities for people with SMI will require similarly multifaceted solutions. Luckily, research has recently been building momentum to address these disparities. We are hopeful that demonstrating cases like his will assist sparking more rapid changes in the direction of coordinated care, combined facilities, and shared communication of medicine and psychiatry developing in the near future. The health system has always been better at treating a solitary problem, but approaching multiple problems concurrently can be more challenging, yet also more cost-effective and beneficial for both the patient’s psychiatric/mental and physical health.

## References

[1] Ghazarian AA, Trabert B, Graubard BI, Schwartz SM, Altekruse SF, McGlynn KA (2015). Incidence of testicular germ cell tumors among US men by census region. Cancer.

[2] Bosl GJ, Motzer RJ (1997). Testicular germ-cell cancer. N Engl J Med.

[3] Aberger M, Wilson B, Holzbeierlein JM, Griebling TL, Nangia AK (2014). Testicular self-examination and testicular cancer: a cost-utility analysis. Cancer Med.

[4] Chapple A, Ziebland S, McPherson A (2004). Qualitative study of men's perceptions of why treatment delays occur in the UK for those with testicular cancer. Br. J. Gen. Pract.

[5] Bosl GJ, Vogelzang NJ, Goldman A (1981). Impact of delay in diagnosis on clinical stage of testicular cancer. Lancet.

[6] Shaw J (2008). Diagnosis and treatment of testicular cancer. Am Fam Physician.

[7] Calonge N (2005). U.S. Preventive Services Task Force Agency for Healthcare Research and Quality. Screening for testicular cancer: recommendation statement. Am Fam Physician.

[8] Lip SZL, Murchison LED, Cullis PS, Govan L, Carachi R (2013). A meta-analysis of the risk of boys with isolated cryptorchidism developing testicular cancer in later life. Arch Dis Child.

[9] (2020). Key substance use and mental health indicators in the United States: Results from the 2017 National Survey on Drug Use and Health. https://store.samhsa.gov/product/Key-Substance-Use-and-Mental-Health-Indicators-in-the-United-States-Results-from-the-2017-National-Survey-on-Drug-Use-and-Health/SMA18-5068.

[10] (2020). Mental Illness. https://www.nimh.nih.gov/health/statistics/mental-illness.shtml.

[11] Bobba VS, Mittal BB, Hoover SV, Kepka A (1988). Classical and anaplastic seminoma: difference in survival. Radiology.

[12] Lavery HJ, Bahnson RR, Sharp DS, Pohar KS (2009). Management of the residual post-chemotherapy retroperitoneal mass in germ cell tumors. Ther Adv Urol.

[13] Atuhaire C, Byamukama A, Cumber RY, Cumber SN (2019). Knowledge and practice of testicular self-examination among secondary students at Ntare School in Mbarara District, South western Uganda. Pan Afr Med J.

[14] Kuzgunbay B, Yaycioglu O, Soyupak B (2013 Apr). Public awareness of testicular cancer and self-examination in Turkey: a multicenter study of Turkish Urooncology Society. Urologic Oncology.

[15] Druss BG (2007). Improving medical care for persons with serious mental illness: challenges and solutions. J Clin Psychiat.

[16] Lawrence D, Kisely S (2010). Inequalities in healthcare provision for people with severe mental illness. J Psychopharmacol.

[17] Heidenreich A, Hofmann Hofmann, R R (1999). Quality-of-life issues in the treatment of testicular cancer. World J Urol.

